# Lipid rescue of massive verapamil overdose: a case report

**DOI:** 10.1186/1752-1947-5-399

**Published:** 2011-08-20

**Authors:** Conrad W Liang, Sarah J Diamond, Daniel S Hagg

**Affiliations:** 1 Department of Neurology, Oregon Health and Science University, 3181 SW Sam Jackson Park Road, Portland, Oregon, 97201, USA; 2 Department of Medicine, Oregon Health and Science University, 3181 SW Sam Jackson Park Road, Portland, Oregon, 97201 USA

## Abstract

**Introduction:**

Massive intentional verapamil overdose is a toxic ingestion which can cause multiorgan system failure and has no currently known antidote.

**Case Presentation:**

The patient is a 41-year-old Caucasian woman who ingested 19.2 g of sustained release verapamil in a suicide attempt. Our patient became hypotensive requiring three high-dose vasopressors to maintain arterial pressure. She also developed acute respiratory failure, bradycardic ventricular rhythm necessitating continuous transvenous pacing, and anuric renal failure. Our patient was treated with intravenous calcium, bicarbonate, hyperinsulinemic euglycemic therapy and continuous venovenous hemodialysis without success. On the fourth day after hospital admission continuous intravenous lipid therapy was initiated. Within three hours of beginning lipid therapy, our patient's vasopressor requirement decreased by half. Within 24 hours, she was on minimal vasopressor support and regained an underlying junctional rhythm. After three days of lipid infusion, she no longer required inotropic agents to maintain blood pressure or pacing to maintain stable hemodynamics.

**Conclusions:**

Intravenous fat emulsion therapy may be an effective antidote for massive verapamil toxicity.

## Introduction

Calcium channel blockers are commonly prescribed for hypertension and rate control of atrial fibrillation, but can be highly toxic in large quantities. Massive overdose of these medications is a serious clinical problem seen rarely in the intensive care unit; management is typically supportive, with variable rates of success [1-4]. Here, we report the successful use of intravenous lipid therapy to reverse the systemic effects of severe verapamil toxicity and we review the literature relating to this complex problem.

Verapamil is a non-dihydropyridine calcium channel blocker with greater cardioselectivity than diltiazem at therapeutic levels. One review suggests that, on overdose, verapamil is more likely to depress atrioventricular (AV) nodal conduction [1]. Peak plasma concentration occurs in up to eight hours in sustained release verapamil, while its half life ranges from two to eight hours and its volume of distribution is 250 to 400 L [5]. Toxic effects common to all calcium channel blockers include dysrhythmias, hypotension and bradycardia from depression of the sinoatrial node. Overdose is rare but frequently lethal. In a 2007 annual report of poison centers, calcium channel blockers accounted for only 12% of all cardiovascular drug exposures but 47% of deaths due to cardiovascular drug poisoning [6]. Reported complications include bowel infarction, stroke, hyperglycemia, and non-cardiogenic pulmonary edema [2]. Massive verapamil overdose, defined here as ingestion of greater than 8 g, is almost uniformly fatal with few case reports of survivors [7-9].

Until recently, there has been no known antidote. Treatment is largely supportive with gastrointestinal decontamination and inotropic support as needed to maintain blood pressure [2,3]. Intravenous calcium infusion is logically indicated to overcome intracellular hypocalcemia but the benefit in overdose is unclear and may even be toxic [10]. Hyperinsulinemic euglycemia (HIE) therapy and glucagon administration have been suggested to improve cardiac inotropy and utilization of glucose, with some success [2,11-13]. However, all of these interventions act secondarily by supporting the body while the drug is naturally eliminated, as opposed to directly neutralizing the circulating drug. In contrast, lipid infusions are thought to sequester lipophilic drugs like verapamil thus, in theory, directly decreasing their bioavailability in the body [14,15].

We present a case of a single-drug, massive verapamil overdose in a failed suicide attempt that was refractory to standard resuscitation techniques, IV calcium and bicarbonate, HIE therapy and continuous venovenous hemodialysis (CVVH), but rapidly responded to intravenous lipid infusion.

## Case Presentation

A 41-year-old Caucasian woman with a history of depression presented to the emergency department (ED) of a referring hospital shortly after taking 80 tablets of sustained release verapamil 240 mg (19.2 g). At the ED, she was treated with multiple doses of activated charcoal, fluids and calcium, and was subsequently transferred to our intensive care unit six hours post-ingestion. Her vital signs on admission were normal (see Table 1 for timeline of our patient's clinical decline and recovery). An initial exam was unremarkable; our patient was easily aroused, alert and oriented, and appeared well. Serum chemistry, complete blood count and liver function tests were normal except for a mildly elevated serum calcium of 10.8 mg/dL and a hematocrit of 36.5%. A urine drug screen was negative, and acetaminophen and salicylate levels were undetectable. Her initial electrocardiogram (ECG) at the referring ED showed normal sinus rhythm, left ventricular hypertrophy, and poor R wave progression. At the time of transfer, the ECG revealed sinus bradycardia and inverted T waves in the anterior precordial leads. Telemetry monitoring demonstrated occasional premature ventricular contractions and apparent U waves. The Oregon Poison Center was consulted at this time, and no specific antidotes were initiated given the patient's clinical appearance. In particular, whole bowel irrigation was not recommended.

**Table 1:** Timeline of the patient's clinical course through the administration of lipids

Time	Temp	Pulse	Rhythm	BP	Vent	Epi	Norepi	Vaso
**Admit** **(Day 1)**	36.7	59	SB	115/73	99% 2L NC	0	0	0

**+8 hrs**		56	Junctional	85/50	91% 6L NC	0	0	0
***Abrupt clinical decline; intubation necessitated; bradycardia requiring transvenous pacer; vasopressors, furosemide, hydrocortisone, antibiotics, calcium, insulin and glucagon infusions begun***								
**+24 hrs** **(Day 2)**	38.5	100	Paced	97/60	SIMV FiO2 80%	0.02	0.3	0.04
***Hypercoagulable requiring heparin infusion; bicarbonate infusion for acidosis; oliguric with renal failure requiring continuous venovenous hemodialysis (CVVH)***								
**+48 hrs** **(Day 3)**	38.2	70	Paced	90/49	SIMV FiO2 85%	0	0.418	0.05
***Worsening respiratory status requiring airway pressure release ventilation (APRV); anuric and pacer dependent***								
**+72 hrs** **(Day 4)**	38.4	70	Paced	124/72	APRV FiO2 80%	0.04	0.75	0.05
***Intravenous lipid infusion started***								
**+76 hrs**	37.5	70	Paced	149/63	APRV FiO2 90%	0.04	0.75	0.05

**+80 hrs**	37.2	70	Paced	128/72	APRV FiO2 50%	0.04	0.3	0.04

**+96 hrs** **(Day 5)**	36.8	70	Paced	134/88	APRV FiO2 50%	0.03	0.1	0.04

**+120 hrs** **(Day 6)**	37.2	70	Paced	106/58	APRV FiO2 50%	0.01	0.05	0
***Improving respiratory status; APRV, CVVH, lipids, HIE, calcium, bicarbonate, and pacing stopped***								
**+144 hrs** **(Day 7)**	37.6	67	Junctional	101/54	Vol A/C FiO2 65%	0	0.03	0

Time measured as hours post-admission, which was six hours post-ingestion. Temperature recorded in Celsius. Rhythm SB: sinus bradycardia; Blood pressure (BP) measured by cuff or arterial line if available; Vent NC: nasal cannula on liters of oxygen; SIMV: synchronized intermittent mandatory ventilation with fraction of inspired oxygen (FiO2); APRV: airway pressure release ventilation with FiO2; Epi: epinephrine in μg/kg/min; Norepi: norepinephrine in μg/kg/min; Vaso: vasopressin in units/min.

Eight hours after admission, at 14 hours post-ingestion, our patient's systolic blood pressure dropped to 80-90 mmHg and her heart rate to 50-60 beats per minute (bpm). An ECG showed third degree AV block. Clinically, our patient became lethargic, her oxygen saturation dropped while on a nasal cannula and she was placed on a 100% non-rebreather. Rapid calcium administration, fluids and dopamine were given with no response; isoproterenol produced a small increase in heart rate. A central venous catheter was placed, and norepinephrine and epinephrine infusions were started. Despite these interventions, our patient's clinical condition continued to worsen. She underwent endotracheal intubation and a temporary transvenous pacemaker was placed to maintain her heart rate. HIE therapy was initiated with 10% dextrose and insulin infusions, with the insulin initially dosed at 40 units/hr with no bolus. Glucagon infusion was started at this time as well. Empiric vancomycin and piperacillin/tazobactam were added due to leukocytosis of 17,400/mm^3^. Calcium chloride infusion was continued throughout this time to maintain an elevated ionized calcium level.

By the next morning, our patient's blood pressure had been stabilized with norepinephrine, epinephrine and vasopressin infusions, and her pulse was maintained by transvenous pacing. Insulin infusion was briefly increased to 60 units/hr before settling at 30 units/hr. She developed simultaneous anion and non-anion gap metabolic acidoses and was started on a bicarbonate infusion. Her chest X-ray and physical exam were consistent with significant volume overload from her multiple infusions and resuscitation, and a furosemide infusion was started. Her central venous pressure at this time ranged from 22 to 46 mmHg. On hospital day three, she developed a thrombosis of her right radial artery at the site of an arterial catheter and was started on intravenous heparin. Her previous urine culture grew > 100,000 colonies of *Staphyloccoccus *with a white count climbing to 31,700/mm^3 ^and fever to 40°C; broad spectrum antibiotics were continued. Our patient subsequently developed refractory hypoxemia and was switched to airway pressure release ventilation (APRV) with supplemental nitric oxide and cisatracurium, with improvement in her oxygenation. She developed anuric renal failure and was started on CVVH. As a result of the initial resuscitation and these interventions, she received a total of 40.7 L of fluid. Despite these therapies, our patient's condition continued to decline. At the nadir of her clinical course, she had no underlying heart rhythm without pacemaker support and required 0.75 μg/kg/hr of norepinephrine, 0.04 μg/kg/hr of epinephrine, and 0.05 units/min of vasopressin to maintain a perfusing mean arterial pressure.

A literature review suggested the use of lipid therapy as a possible antidote, and on hospital day four our patient was started on an intralipid infusion with a 100 ml bolus of 20% followed by continuous infusion at 0.5 ml/kg/hr. Three hours after the infusion was started, the norepinephrine dose was decreased by more than 50% to 0.3 μg/kg/hr. After a further 48 hours, vasopressin had been stopped and her vasopressor requirement had dropped to 0.01 μg/kg/hr of epinephrine and 0.05 μg/kg/hr of norepinephrine. On hospital day six, our patient regained a junctional heart rhythm with a heart rate of 65-70 bpm and transvenous pacing was discontinued. Her respiratory status had also improved significantly enough to stop APRV, nitric oxide and cisatracurium. On day seven, she was weaned off vasopressors and changed to pressure support ventilation. Her renal function improved, urine output increased, and CVVH was discontinued. At this point, it was felt that she had overcome the acute effects of verapamil overdose, and her calcium, HIE therapy and intravenous lipids were discontinued. Her blood pressure normalized and her heart rhythm was sinus tachycardia with a rate into the 110s. A total of 4,200 mL of intralipid was administered over the initial seven days in the intensive care unit.

Our patient's recovery was complicated by leukocytosis and tachycardia that persisted even after her blood pressure and renal failure had improved. She had a distended abdomen, but was able to communicate and follow simple commands while intubated and did not complain of significant abdominal discomfort. A computed tomography scan of her abdomen revealed pneumatosis intestinalis, and our patient underwent a same-day, urgent subtotal colectomy and colostomy for ischemic colitis. She slowly improved and was discharged from the intensive care unit on 33 days after ingesting the tablets. She was discharged from the hospital on post-ingestion day 55 to a skilled nursing facility. At discharge she was alert and oriented with intact verbal function, was able to walk with standby assistance, and was eager to be discharged. At the time of publication she has completed extensive physical rehabilitation and will soon be returning home to be with her family.

## Discussion

Over the past decade, intravenous fat emulsion therapy has gained credibility as an antidote for local anesthetic toxicity [16]. It has been a true bench-to-bedside story, emerging from the theory of sequestering lipophilic drugs to successful animal trials and human case reports [17-21]. Focus is now turning to the use of lipid emulsions as successful antidotes for other lipophilic drugs, such as verapamil, beta blockers, and tricyclic antidepressants. Most of these studies have been performed in animal models, and case reports are now appearing to validate the laboratory findings [16]. Despite the lack of large trials, the anecdotal successes are compelling and the indications and limitations for lipid therapy are now being explored [22-24].

Several studies have demonstrated the efficacy of fat emulsion therapy in verapamil overdose in animals [14,15,25]. The initial study by Tebbutt *et al. *showed a dramatic effect, with lipid therapy nearly doubling the time to death, the mean lethal dose and the median lethal dose (LD50) of rodents being infused with verapamil, as compared with saline [14]. Bania *et al. *then demonstrated in a canine model that lipid infusion combined with standard resuscitation techniques led to increased survival, characterized by increased mean arterial pressures detectable within 30 minutes of starting the lipid infusion. At 60 minutes, there was a significant improvement in maximal ventricular pressure, and an observed but non-significant improvement in heart rate, cardiac output, and systemic vascular resistance. At 120 minutes, the survival percentage of animals receiving lipid therapy was 100%, compared to 14% survival of controls receiving saline [25]. A follow-up study in rodents showed that the greatest survival benefit with bolus dosing occurred at 18.6 ml/kg, while the greatest benefit to heart rate, mean arterial pressure, and base excess occurred at 24.8 ml/kg. These doses are higher than the recommended maximal daily dose of 10 ml/kg/day used for artificial parenteral nutrition in humans, which suggests that high dosing may be necessary to reverse severe drug toxicity [15].

Three recent cases illustrate the potential of lipid therapy in calcium channel blocker overdose. Young *et al. *present a case of multidrug overdose including 13.44 g of verapamil in combination with bupropion, zolpidem, quetiapine, clonazepam and benazepril [26]. Their patient presented to the hospital hypotensive and acidotic, requiring mechanical ventilation and norepinephrine to maintain arterial pressure. Lipid therapy was initiated almost immediately upon arrival, and within an hour the norepinephrine dose was halved. The patient was extubated on day two, and discharged on day five with no neurologic deficits. Montiel *et al. *describe a second case of a patient who ingested 3.6 g of sustained-release diltiazem who was treated with hyperinsulinemic euglycemia therapy in combination with lipid therapy [27]. Their patient had an improvement in blood pressure within one hour of beginning intravenous lipid, and was discharged from the intensive care unit on day nine with a full recovery. Finally, French *et al. *report a case of sustained verapamil overdose treated with lipid emulsion therapy. Notably, the serum level of verapamil in their case was slightly decreased after administration of lipids [28].

This is the second report of intralipid fat emulsion therapy being used in verapamil overdose, both in conjunction with high dose insulin and having a benefit after HIE has failed. Significantly, intralipid fat emulsion therapy was given for a longer period than in prior reports, and in response to a much larger overdose of verapamil. Our case demonstrates that lipid therapy can be effective in a pure, massive overdose of sustained-release verapamil, and that this efficacy can manifest several days after ingestion. Our patient was initially managed conservatively with standard resuscitation techniques and care was escalated as her clinical condition declined; lipid therapy was started as it became clear that she was worsening despite our best treatment. Although a causal relationship between lipid therapy and clinical improvement cannot be definitively determined, the time course of her recovery is compelling. Within hours of starting the lipid therapy, her disease course abruptly reversed, and within days she had regained an intrinsic heart rhythm and no longer required pacing or vasopressors. In retrospect, her need for additional therapies, including transvenous pacing, high-dose vasopressors and/or inotropes, HIE therapy, bicarbonate infusion, broad spectrum antibiotics and continuous hemodialysis, may have been lessened if lipid therapy had been started earlier.

The optimal dose, timing, and duration of therapy remain unclear. Animal studies of lipid infusion with verapamil toxicity have used primarily single bolus dosing, while case reports commonly include an initial bolus plus continuous infusion. Our initial dosing was based on previous case reports [26], and the duration of therapy was determined by our patient's clinical improvement. In general, however, intravenous fat emulsions have been given safely and routinely to critically ill patients as parenteral nutrition. Adverse effects in the acute period include allergic reaction and the potential to develop fat or pulmonary emboli. Patients requiring long-term use may also develop liver dysfunction.

## Conclusion

Intravenous lipid therapy is a plausible and effective therapy for massive calcium channel blocker overdose, and could be considered early in patients presenting with hemodynamic compromise.

### Consent

Written informed consent was obtained from the patient for publication of this case report and any accompanying data. A copy of the written consent is available for review by the Editor-in-Chief of this journal.

## Competing interests

The authors declare that they have no competing interests.

## Authors' contributions

CL performed a chart review and literature search. All authors read and approved the manuscript.
